# Evaluating the influence of anti-PD-1 immunotherapy combined with IMRT on thyroid dysfunction in nasopharyngeal carcinoma

**DOI:** 10.3389/fimmu.2024.1495946

**Published:** 2025-01-16

**Authors:** Liqianqi Chen, Zihuang Li, Xianming Li, Shihai Wu, Long Gong, Gang Xu, Shanyi Chen, Yucong Zhang

**Affiliations:** The Second Clinical Medical College of Jinan University, Department of Radiotherapy, Shenzhen People’s Hospital, Shenzhen, Guangdong, China

**Keywords:** PD-1, radiotherapy, thyroid dysfunction, nasopharyngeal carcinoma, IMRT (intensity modulated radiation therapy)

## Abstract

**Background:**

Immunotherapy represents a major breakthrough in malignant tumor treatment in recent years. Anti-PD-1 immunotherapy has significantly prolonged Event-free Survival (EFS) in Nasopharyngeal Carcinoma (NPC). However, its potent anti-tumor effects can also attack normal tissues and organs, leading to immune-related adverse effects (irAE), with the thyroid being one of the most commonly affected organs. This study aims to analyze the incidence and related factors of thyroid dysfunction in NPC patients receiving anti-PD-1 immunotherapy with/without Intensity-modulated radiotherapy (IMRT), and further explore whether radiotherapy interacts with thyroid immune-related adverse reactions.

**Methods:**

108 NPC patients receiving immunotherapy combined with chemotherapy or chemoradiotherapy were retrospectively included. Data collected included smoking status, BMI, presence of thyroid nodules, staging, treatment modality, thyroid mean dose (Dmean), percentage of thyroid volume receiving more than x Gy, pituitary mean dose (Dmean), and TSH and FT4 levels per cycle. T-tests, rank-sum tests, multivariate logistic regression analysis, ROC curves, and Cox proportional hazards models were used to evaluate the effects of anti-PD-1 immunotherapy combined with chemoradiotherapy on thyroid function.

**Results:**

Patients with pre-treatment smoking history, thyroid nodules, and cervical lymph node metastasis were more likely to develop thyroid dysfunction (P<0.05). During treatment, 81 patients developed varying degrees of thyroid dysfunction. Subclinical hyperthyroidism (33.9%) was most common in the immunotherapy plus chemoradiotherapy group, while subclinical hypothyroidism (23.9%) was most common in the immunotherapy plus chemotherapy group. Compared to the immunotherapy plus chemotherapy group, the immunotherapy plus chemoradiotherapy group showed higher incidence and severity of hyperthyroidism (median peak FT4 concentration: 19.11 pmol/L vs 16.21 pmol/L) (P=0.001). The immunotherapy plus chemoradiotherapy group showed lower incidence but increased severity of hypothyroidism compared to the immunotherapy plus chemotherapy group, though these differences were not statistically significant.

**Conclusion:**

NPC patients with smoking history, thyroid nodules, and cervical lymph node metastasis have significantly increased risk of thyroid dysfunction when receiving anti-PD-1 immunotherapy combined with IMRT. The combination of anti-PD-1 immunotherapy and IMRT increases both the incidence and severity of thyroid dysfunction.

## Introduction

Nasopharyngeal carcinoma (NPC) is an epithelial cancer originating in the nasopharyngeal mucosa. According to the International Agency for Research on Cancer, there were approximately 133,000 new cases and 80,000 deaths from NPC in 2020, with China accounting for 47% of global new cases (1). Radiotherapy is the primary treatment for NPC, with concurrent chemoradiotherapy or combined neoadjuvant chemotherapy considered the standard treatment for locally advanced NPC. Despite these intensive treatment approaches, 20%-30% of patients still experience disease recurrence (2–4), resulting in suboptimal survival outcomes.

Immunotherapy has emerged as a major breakthrough in cancer treatment in recent years. Immune checkpoint inhibitors (ICIs) have significantly extended progression-free survival (PFS) and overall survival (OS) in recurrent/metastatic NPC. According to the 2022 NCCN guidelines (4), PD-1 inhibitors are now recommended as first-line and second-line treatment options for recurrent/metastatic NPC. Additionally, clinical trials investigating first-line treatment (pre-, during, or post-radiotherapy) for non-recurrent/metastatic NPC are widely ongoing, showing promising preliminary results (5).

As one of the most widely used immunotherapy drugs, PD-1 inhibitors may trigger autoimmune responses in multiple systems, including the endocrine system, due to excessive immune cell activation. These reactions are known as immune-related adverse events (irAEs). Endocrine dysfunction is among the most common adverse events reported in ICI clinical trials, including hypothyroidism, hyperthyroidism, pituitary inflammation, primary adrenal insufficiency, and insulin resistance, with thyroid-related immune adverse events being the most frequent (6).

Due to NPC high radiosensitivity, radiotherapy has long been the preferred treatment method. During radiotherapy for NPC patients, the thyroid gland is often partially or completely included in the radiation field due to its unique anatomical position. This results in high-dose radiation exposure, leading to various early and late reactions and causing multiple acute and chronic functional abnormalities, primarily manifesting as hypothyroidism, including both subclinical and clinical cases. These thyroid dysfunctions can cause various discomforts, affecting physical health and, in severe cases, significantly reducing quality of life. While maintaining tumor coverage, the thyroid typically receives doses exceeding 50Gy, easily causing damage (7). The median time to hypothyroidism after intensity-modulated radiotherapy is 1.4-1.8 years, with an incidence rate of 23%-53% (8). Under current radiation techniques, most tumor and normal tissue cell death occurs through mitotic death, where cells may undergo several divisions before dying, explaining why thyroid dysfunction may manifest long after completing radiotherapy.

Current research generally attributes radiation-induced hypothyroidism to four main mechanisms: 1. Direct radiation damage to thyroid cells, causing DNA strand damage and subsequent cell death (9). 2. Vascular damage to the thyroid, where ionizing radiation increases apoptosis of vascular endothelial and smooth muscle cells, inhibits proliferation, and triggers inflammatory responses through various cytokines, mediators, and inflammatory cells (10, 11). 3. Thyroid capsule fibrosis, preventing compensation for thyroid cell damage and leading to atrophy and chronic inflammation (8). 4. Pituitary damage affecting thyroid hormone regulation, as the radiation field may include part of the pituitary gland in addition to the thyroid. While post-radiotherapy thyroid dysfunction primarily presents as hypothyroidism, some cases manifest as hyperthyroidism. The mechanisms for hyperthyroidism may include:1. Direct thyroid tissue damage causing inflammation or other pathological changes, leading to excessive thyroid hormone secretion. 2. Impact on the immune system causing autoimmune responses, such as the production of thyroid autoantibodies. 3. Structural changes in thyroid tissue affecting thyroid hormone regulation.

With increasing use of anti-PD-1 immunotherapy in NPC treatment, experts are focused on whether radiotherapy worsens immunotherapy-induced thyroid dysfunction and if there’s synergy between them. No consensus exists yet. This study analyzes thyroid function changes in 108 NPC patients receiving chemo-immunotherapy ± radiotherapy to provide clinical guidance.

## Methods and materials

A retrospective study of 108 NPC patients treated at Shenzhen People’s Hospital between January 2019 and August 2024. Metastatic patients received chemotherapy plus immunotherapy (immunotherapy group), while non-metastatic/recurrent patients received concurrent chemoradiotherapy plus immunotherapy (combined therapy group). The median follow-up was 152 days, with immunotherapy given in 21-day cycles for 7 total cycles, and a median of 33 radiation fractions.

Inclusion criteria: Patients aged 25-70 (median 42), including 76 males and 32 females, with pathologically confirmed NPC (WHO type: undifferentiated non-keratinizing squamous cell carcinoma), willing to receive radiotherapy and PD-1 immunotherapy, and PS score 0-2. Exclusion criteria: pre-existing thyroid dysfunction or related diseases/surgeries, previous head/neck radiation or immunotherapy, hypothalamic-pituitary axis disorders, and incomplete clinical data. Clinical staging followed AJCC 8th edition TNM system.

The 108 patients were divided into two groups based on treatment modality: immunotherapy group (immunotherapy plus chemotherapy) and combined therapy group (immunotherapy plus chemoradiotherapy). Based on thyroid function changes, patients were further categorized into hyperthyroid, hypothyroid, and no-change groups.

Observation parameters: Demographic data (age, sex, smoking status, BMI), thyroid nodule status, staging, and treatment modality. Treatment types included chemotherapy, radiotherapy, and immunotherapy. Radiotherapy parameters measured included thyroid mean dose (Dmean), percentage of thyroid volume receiving >x Gy [Vx(%) for x=35-60 in 5Gy increments], and pituitary mean dose. Thyroid function was assessed through TSH (most sensitive early indicator of dysfunction) and FT4 (most sensitive for hypothyroidism diagnosis) blood levels.

Study endpoint was defined as thyroid function assessment after the 7th immunotherapy cycle. Time of onset was measured from first ICI dose to initial thyroid dysfunction. Thyroid dysfunction was classified as clinical hyperthyroidism (low TSH with elevated FT4), subclinical hyperthyroidism (low TSH with normal FT4), clinical hypothyroidism (high TSH with low FT4), or subclinical hypothyroidism (high TSH with normal FT4). Patients experiencing transient hyperthyroidism before developing hypothyroidism were classified in the corresponding hypothyroid group. Laboratory reference ranges were set at TSH: 0.56-5.91 mIU/L and FT4: 97.98-16.02 pmol/L.

Radiotherapy setup: Patients were positioned supine with thermoplastic head-neck-shoulder mask fixation from skull vertex to shoulders, using 4-5 point fixation. Enhanced CT scanning (3mm slices) ranged from skull vertex to 2cm below clavicular head. Target volumes were contoured using MRI reference: GTVnx (primary tumor and retropharyngeal nodes), GTVnd (positive neck nodes), CTV1 (high-risk areas), CTV2 (low-risk areas). Post-neoadjuvant therapy GTVnx was based on pre-treatment MRI, GTVnd on post-treatment MRI. CTVs had 5mm margins, PTVs 3-5mm (reduced to 1-2mm near critical structures). Prescribed doses: PGTVnx/nd 68-70Gy, PCTV1 60-64Gy, PCTV2 54-58Gy/30-33F. Organ constraints included thyroid Dmean ≤ 45Gy. Treatment delivered via IMRT (Eclipse) using 6MV X-ray linear accelerator (VARIAN), median 33 fractions. Thyroid contouring and treatment planning followed the 2017 international guidelines for nasopharyngeal cancer target delineation (12). Thyroid dose parameters including volume, maximum dose, minimum dose, and mean dose were precisely calculated.

All 108 nasopharyngeal cancer patients received platinum monotherapy chemotherapy combined with anti-PD-1 immunotherapy concurrent with radiotherapy.

All patients received anti-PD-1 immunotherapy (as shown in **Table 1**) administered every three weeks until disease progression or intolerable toxicity, to control for potential variations in outcomes among different types of immune checkpoint inhibitors (ICIs) such as PD-1, PD-L1, and CTLA-4.

**Table 1:** PD-1 drugs of all types.

Immunodrug	N	Proportion
Sintilimab	55	50.9%
Toripalimab	28	25.9%
Tislelizumab	14	13.0%
Pembrolizumab	7	6.5%
Nivolumab	4	3.7%

Statistical analysis utilized SPSS 26.0 software. Count data were expressed as numbers and percentages. Normally distributed continuous variables were described as mean ± standard deviation, while non-normally distributed variables used interquartile ranges. Mann-Whitney U and Kruskal-Wallis tests were used for group comparisons. Multivariate logistic regression analysis and ROC curves were used to analyze clinical and biochemical characteristics of thyroid dysfunction. Statistical significance was set at P<0.05.

## Results

Study included 108 NPC patients with median age 42 years at first ICI treatment, 70.4% male. Thyroid dysfunction distribution: 54 developed hyperthyroidism, 27 hypothyroidism, and 27 remained unchanged. In the combined therapy group (n=62): 9 (14.5%) developed hypothyroidism, 38 (61.3%) hyperthyroidism, and 15 (24.2%) no change. In immunotherapy-only group (n=46): 18 (39.1%) developed hypothyroidism, 16 (34.8%) hyperthyroidism, and 12 (26.1%) no change. Thyroid dysfunction was significantly associated with treatment modality (P<0.05). Baseline thyroid nodules correlated with hyperthyroidism development (P<0.05, **Table 2**), while N staging influenced hypothyroidism occurrence (P<0.05, **Table 3**).

**Table 2:** Baseline characteristics of patients with hyperthyroidism.

	hyperthyroidism group (n=54)	Non- hyperthyroidism group (n=54)	P value
Gender			1.000
Male	38	38	
Female	16	16	
Age			.060
<40 years	27	15	
~ 59 years	22	32	
ge; 60 years	5	7	
Smoke			.071
No	15	24	
Yes	39	30	
BMI			.479
<18.5 kg/m2	9	13	
18.5 ~ 23.9 kg/m2	36	30	
≥ 24 kg/m2	9	11	
Thyroid nodule			.001
No	12	29	
Yes	42	25	
T staging			.318
1	4	2	
2	10	8	
3	25	20	
4	15	24	
N staging			.960
0	0	0	
1	9	8	
2	22	23	
3	23	23	
M staging			.069
0	40	30	
1	14	21	
Treatment mode			.011
R+C+I ^a^	38	24	
C+I ^b^	16	30	

^a^Radiotherapy +Chemotherapy + Immunotherapy. ^b^Chemotherapy +Immunotherapy.

**Table 3:** Baseline characteristics of patients with hypothyroidism.

	hypothyroidism group (n=27)	Non- hypothyroidism group (n=81)	P value
Gender			.808
Male	20	56	
Female	7	25	
Age			.125
<40 years	8	34	
40 ~ 59 years	13	41	
≥ 60 years	6	6	
Smoke			.107
No	6	33	
Yes	21	48	
BMI			.734
<18.5 kg/m2	4	17	
.5 ~ 23.9 kg/m2	17	50	
≥ 24 kg/m2	6	14	
Thyroid nodule			.651
No	9	32	
Yes	18	49	
T staging			.910
1	2	4	
	4	14	
3	11	35	
4	10	28	
N staging			.015
	0	0	
1	0	16	
2	16	31	
3	11	34	
M staging			.061
0	13	57	
1	14	24	
Treatment mode			.006
R+C+I ^a^	9	53	
C+I ^b^	18	28	

^a^Radiotherapy + Chemotherapy + Immunotherapy. ^b^Chemotherapy + Immunotherapy.

Multivariate analysis of post-treatment hyperthyroidism in NPC patients identified smoking, pre-treatment thyroid nodules, and N staging as significant risk factors (P<0.05), as shown in **Table 4**.

**Table 4:** Multifactorial logistic regression analysis of hyperthyroidism.

	B	S.E.	Wald	P	Exp(B)
Gender	-.310	1.263	.060	.806	.733
Age	.534	.764	.488	.485	1.705
Smoke	-2.519	.942	7.149	.008	.081
Weight	.006	.067	.009	.923	1.006
BMI	-1.100	1.224	.808	.369	.333
Thyroid nodule	-2.483	1.015	5.980	.014	.084
T	.197	.650	.092	.762	1.217
N	-3.641	1.065	11.700	.001	.026
M	1.483	1.107	1.793	.181	4.405
PD-1	.500	.474	1.114	.291	1.650
Constant	8.440	5.283	2.552	.110	4627.930

**Figure 1:**
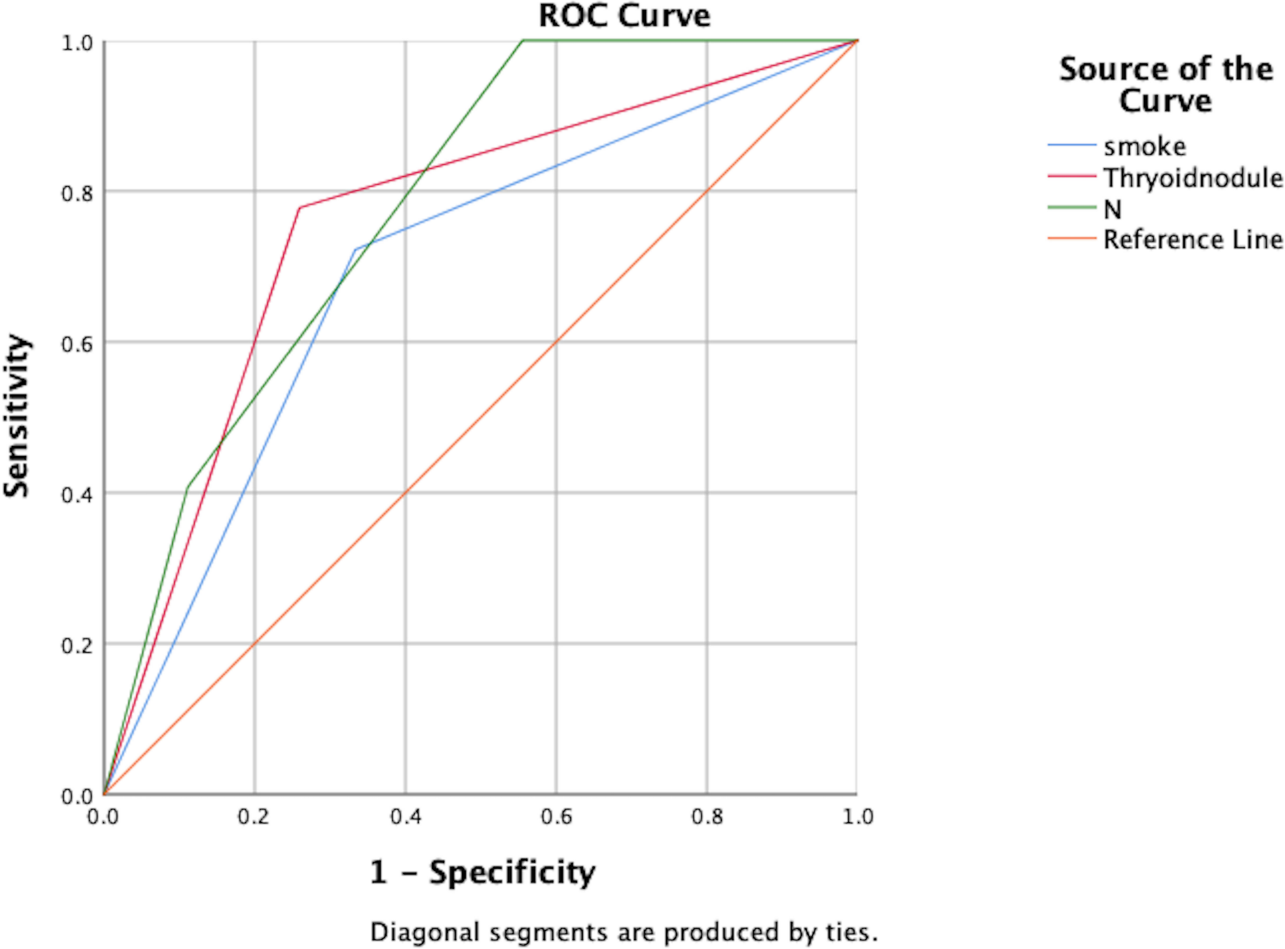
ROC analysis of the predictive value of smoking, thyroid nodules, and N-staging for the development of hyperthyroidism.

Independent risk factors for post-treatment hyperthyroidism in NPC patients were identified through multivariate analysis: smoking, pre-treatment thyroid nodules, and N staging. ROC curve analysis showed AUC values of 0.694, 0.759, and 0.780 respectively, indicating good predictive capability for these risk factors (P<0.05), as shown in **Figure 1**; **Table 5**.

**Table 5:** AUC values for each factor of hyperthyroidism.

	AUC	S. E.	P	95% CI	
				Lower Bound	Upper Bound
smoke	.694	.064	.005	.570	.819
Thyroid nodule	.759	.059	.000	.644	.875
N	.780	.058	.000	.666	.894

Multivariate analysis of post-treatment hypothyroidism in NPC patients identified smoking, pre-treatment thyroid nodules, and N staging as significant risk factors (P<0.05), as shown in **Table 6**.

**Table 6:** Multifactorial logistic regression analysis of hypothyroidism.

	B	S.E.	Wald	P	Exp(B)
Gender	-.080	1.103	.005	.942	.923
Age	-.017	.043	.156	.692	.983
Smoke	1.883	.832	5.128	.024	6.575
Weight	.137	.109	1.571	.210	1.147
BMI	-.395	.347	1.296	.255	.674
Thyroid nodule	2.064	.861	5.744	.017	7.876
T	.093	.586	.025	.874	1.098
N	1.450	.695	4.357	.037	4.265
M	.306	.905	.114	.735	1.358
PD-1	-.109	.298	.134	.715	.897
constant	-4.064	5.542	.538	.463	.017

**Figure 2:**
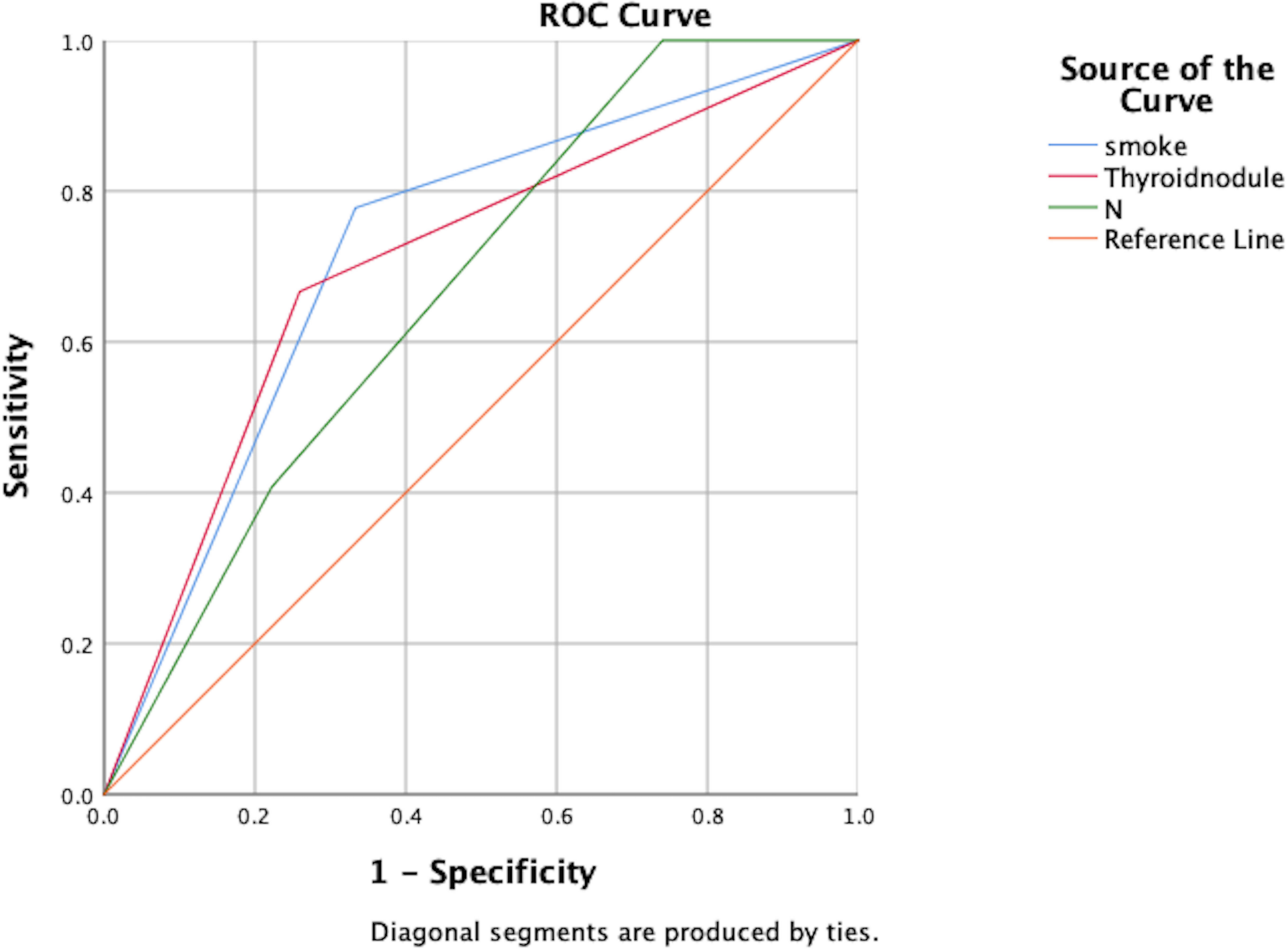
ROC analysis of the predictive value of smoking, thyroid nodules, and N-staging for the development of hypothyroidism.

Independent risk factors for post-treatment hypothyroidism in NPC patients were identified through multivariate analysis: smoking, pre-treatment thyroid nodules, and N staging. ROC curve analysis showed AUC values of 0.722, 0.704, and 0.669 respectively, indicating good predictive capability for these risk factors (P<0.05), as shown in **Figure 2**; **Table 7**.

**Table 7:** AUC values for each factor of hypothyroidism.

	AUC	S. E.	P	95% CI	
				Lower Bound	Upper Bound
smoke	.722	.071	.005	.583	.861
Thyroid nodule	.704	.072	.010	.562	.846
N	.669	.074	.033	.525	.814

**Figure 3:**
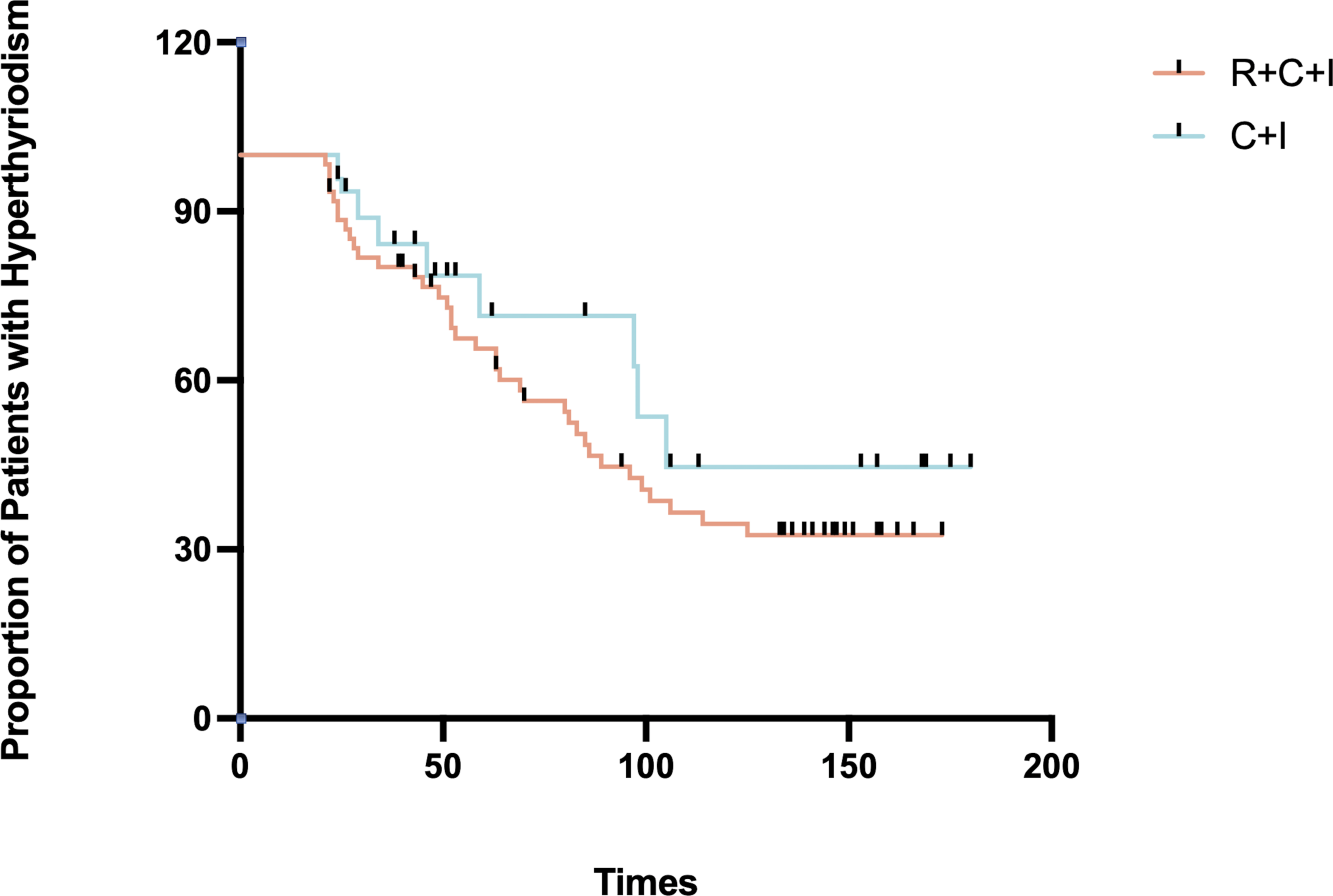
Time to hyperthyroidism in the immunotherapy combined with radiotherapy group versus the immunotherapy alone group.

**Figure 4:**
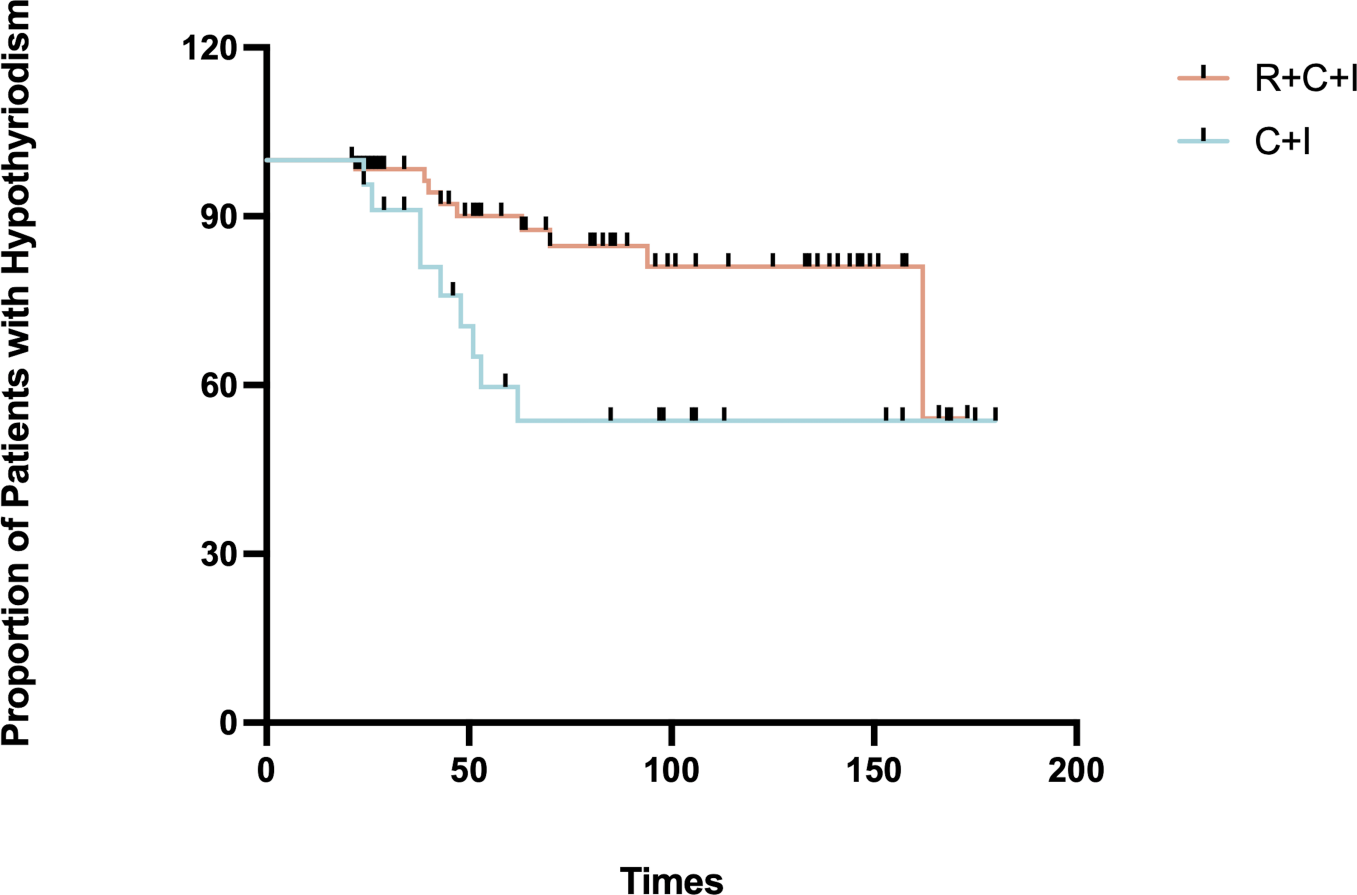
Time to onset of hypothyroidism in the immunotherapy combined with radiotherapy group vs. immunotherapy alone group.

**Figure 5:**
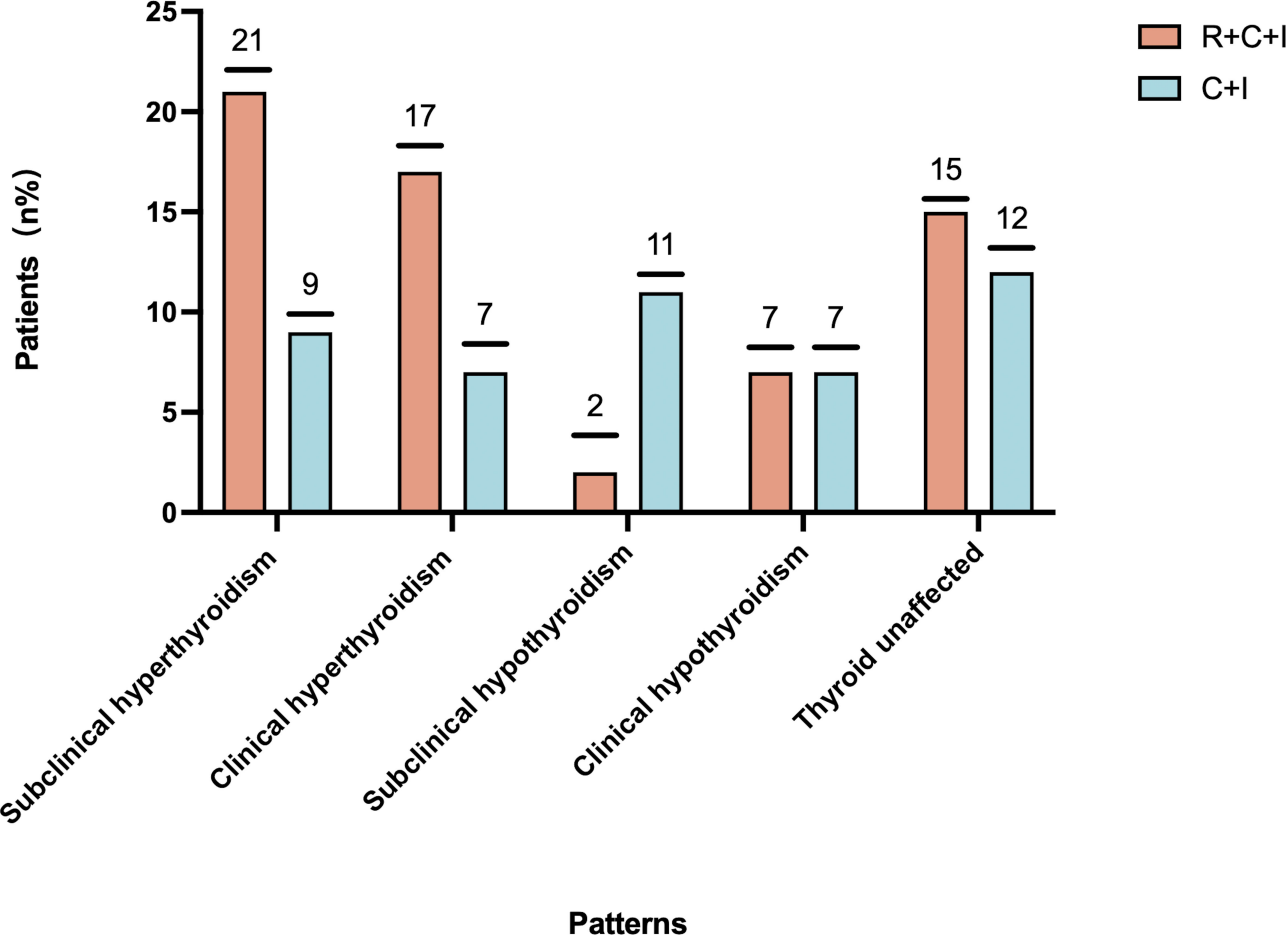
Patterns and incidence of thyroid dysfunction.

Median time to thyroid dysfunction onset was 49 days overall - 50 days in combined therapy group and 46 days in immunotherapy-only group. Hyperthyroidism developed earlier in combined therapy group compared to immunotherapy group (85d vs 105d), though difference was not statistically significant (P>0.05), as shown in **Figures 3**, **4**. Various patterns of thyroid dysfunction were observed: hyperthyroidism was most common (54 patients, 50%), while hypothyroidism and no change each affected 27 patients (25%). In combined therapy group (n=62), 38 cases (61.3%) developed hyperthyroidism (21 subclinical, 17 clinical) and 9 cases (14.5%) developed primary hypothyroidism, mostly clinical cases. In immunotherapy-only group (n=46), 16 cases (34.8%) developed hyperthyroidism (9 subclinical, 7 clinical) and 18 cases (39.1%) developed hypothyroidism (11 subclinical, 7 clinical), as illustrated in **Figure 5**.

**Figure 6:**
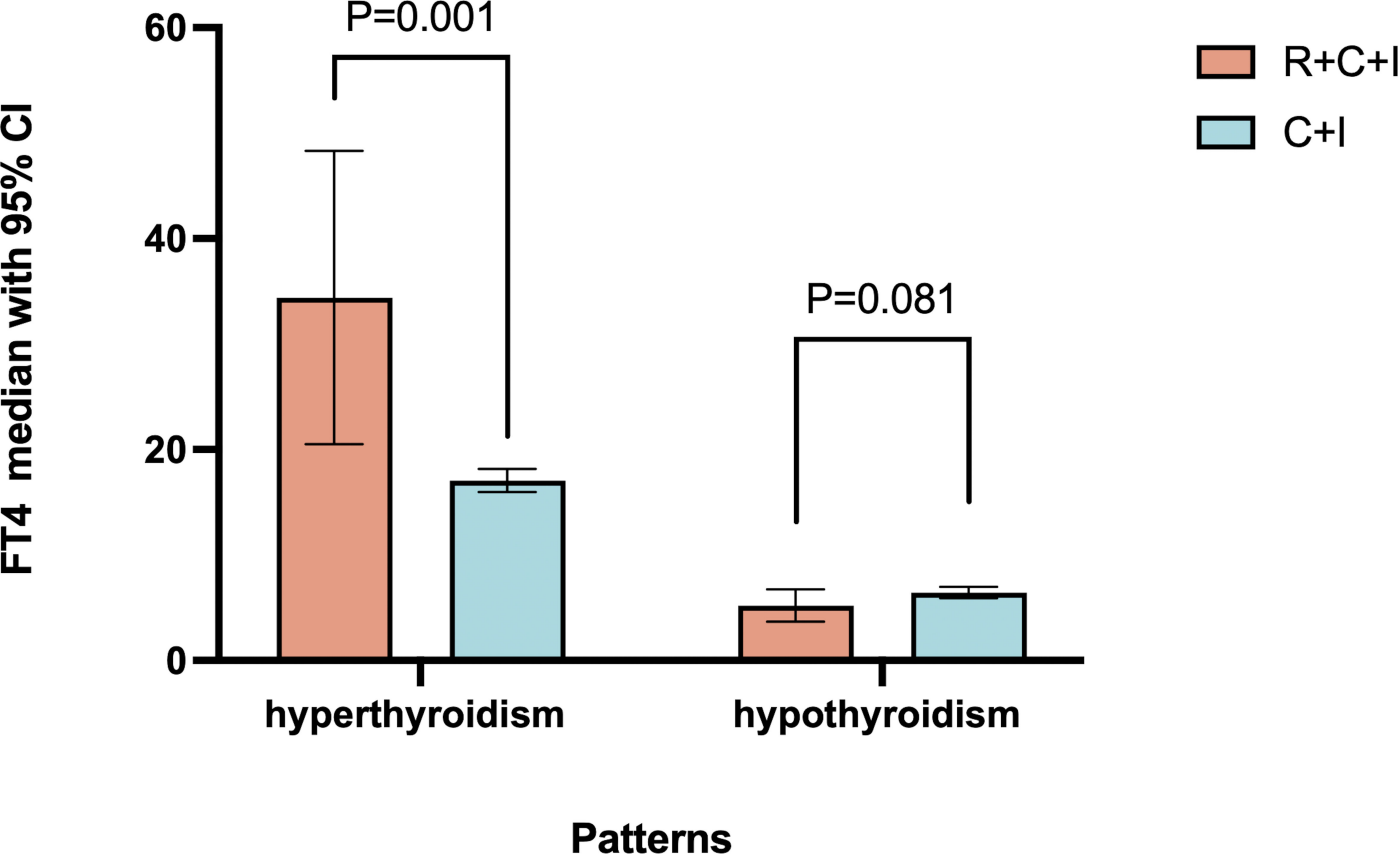
FT4 comparison of abnormal thyroid function.

Among hyperthyroid patients, the immunotherapy group showed median peak FT4 of 16.21 pmol/L (1.2% increase, mean rank 9.5), while the combined therapy group showed median peak FT4 of 19.11 pmol/L (19.3% increase, mean rank 21.4, 55.6% higher than immunotherapy group), showing significant difference (P=0.001) as shown in **Figure 3**. For hypothyroid patients, the immunotherapy group had median lowest FT4 of 6.47 pmol/L (18.9% decrease, mean rank 24.37), while the combined therapy group showed median lowest FT4 of 5.835 pmol/L (26.9% decrease, mean rank 18.5, 24.1% lower than immunotherapy group), though difference was not significant (P=0.081) as shown in **Figure 6**. Four patients progressed from subclinical hyperthyroidism to clinical hypothyroidism during treatment.

## Discussion

Recent years have shown improved NPC patient prognosis through combined radiotherapy and immunotherapy. Growing preclinical and clinical data support the combination of radiotherapy and immune checkpoint inhibitors (13, 14). Studies suggest ICI-related thyroid dysfunction correlates with longer progression-free survival (PFS) and overall survival (OS) (15, 16). In this short-term follow-up study, thyroid dysfunction incidence was 31.2% in the immunotherapy group, slightly lower than current largest cohort studies of ICI-related thyroid irAE, with potential for increased incidence during longer follow-up. The combined therapy group showed significantly higher thyroid dysfunction incidence at 43.5%. This study aimed to compare clinical and biochemical characteristics of thyroid dysfunction after combined radiotherapy and immunotherapy, suggesting potential heterogeneity between clinical and subclinical thyroid dysfunction occurrence.

In this study, smoking, thyroid nodules, and N staging were identified as risk factors for thyroid dysfunction. Tobacco, containing nicotine and thiocyanate, interferes with TSH levels, thyroid hormone metabolism, and immune system function, promoting thyroid dysfunction during NPC treatment. Non-functioning thyroid nodules may develop secretory function after radiation exposure or PD-1 inhibitor treatment, affecting thyroid hormone synthesis and release, leading to thyroid dysfunction. Lymph node positivity is a factor influencing radiation-induced hypothyroidism in head and neck cancer radiotherapy patients (17). NPC patients with N2-3 vs N0-1 patients (37.38% vs 13.11%) (18). In a large cohort study, gender and age were strong predictors of thyroid dysfunction (19). However, our study did not find such significance, possibly due to small sample size and lack of statistical power.

Several studies have demonstrated that mean thyroid radiation dose is an independent risk factor for radiation-induced hypothyroidism (13, 20). The threshold dose for radiation-induced thyroid secretory dysfunction is 30Gy, with doses above 30Gy generally considered sufficient to cause thyroid secretory damage (21). In this study, the mean thyroid dose was 49.63 ± 2.69Gy (**Table 8**). Research has shown that patients with the entire thyroid gland included in the target area have significantly higher rates of radiation-induced hypothyroidism compared to those with partial thyroid irradiation (22). Many scholars believe there is a linear relationship between thyroid dose-volume and radiation-induced hypothyroidism. Research has shown that V25 ≤ 60%, V35 ≤ 55%, and V45 ≤ 45% are independent risk factors for predicting radiation-induced hypothyroidism (23). Studies have reported that pituitary radiation doses exceeding 45Gy can cause pituitary damage (24). In this cohort study, the mean pituitary radiation dose was 32.41 ± 6.39Gy (**Table 8**).

**Table 8:** Irradiation dose in the combined treatment group.

	Dose(Gy)
Thyroid Dmean	49.6257 ± 2.69013
Thyroid V35	52.1248 ± 2.11599
Thyroid V40	51.0544 ±2.29639
Thyroid V45	50.0852 ±2.60207
Thyroid V50	49.2881 ±2.77693
Thyroid V55	46.7633 ±4.27295
Thyroid V60	41.3580 ±6.49687
Pituitary Dmean	32.4095 ±6.39475

Thyroid dysfunction following ICI treatment can appear within weeks to months after the first dose, currently understood as immune-mediated destructive thyroiditis, typically presenting as transient hyperthyroidism followed by hypothyroidism. Clinical manifestations of thyroid irAE are diverse, including not only thyroiditis-like presentations but also isolated thyrotoxicosis, hypothyroidism, and subclinical thyroid dysfunction (6). ICI-related thyroid dysfunction manifests as hypothyroidism and hyperthyroidism, with thyroiditis and thyroid storm being relatively rare, and no large-scale studies have reported on these yet. ICI-related hyperthyroidism mainly presents with hypermetabolic symptoms, while ICI-related hypothyroidism shows similar manifestations to conventional hypothyroidism, characterized by decreased metabolic rate and reduced sympathetic nervous system activity.

Radiation-induced thyroid dysfunction presents similarly to conventional hypothyroidism, classified as subclinical or clinical based on severity. Clinical hypothyroidism shows decreased metabolic rate and reduced sympathetic nervous activity, with typical symptoms including fatigue, weakness, cold intolerance, constipation, depression, slow reactions, and dull expressions. Radiation-induced hypothyroidism can occur as early as 1.9 months post-radiotherapy, with incidence reaching 40-50% within 2 years. Subclinical hypothyroidism predominates (approximately 70%) (25, 26) and may progress to clinical hypothyroidism, completely resolve, or remain stable (27). Radiation-induced hyperthyroidism is relatively rare, with an incidence of only 0.6%, and is typically self-limiting. Its clinical symptoms are identical to conventional hyperthyroidism, presenting with hypermetabolic symptoms due to excessive thyroid hormone in circulation.

The earlier onset and increased severity of hyperthyroidism in radiochemotherapy combined with immunotherapy compared to immunotherapy with chemotherapy alone may involve multiple factors. Immune checkpoint inhibitors activate the immune system against cancer cells but can trigger autoimmune side effects, while radiotherapy induces thyroid-specific autoantibodies and enhances systemic immune response, increasing the probability of immune attacks on thyroid tissue. Through high-energy radiation’s direct cell-killing effects, structural changes occur in irradiated thyroid regions, disrupting endocrine function and leading to excessive hormone secretion, which amplifies immunotherapy-induced hyperthyroidism. Additionally, radiotherapy triggers inflammatory cytokine storm in thyroid tissue, releasing pro-inflammatory cytokines like IL-6 and TNF-α, which enhance the systemic immune inflammatory response from immunotherapy. These mechanisms work synergistically, resulting in increased systemic immune activation, enhanced inflammatory responses, and individual variations in thyroid radiation dose, ultimately leading to more severe hyperthyroidism.

The increased severity of hypothyroidism in radiochemotherapy combined with immunotherapy compared to immunotherapy with chemotherapy alone can be attributed to several factors. Radiotherapy for nasopharyngeal cancer can directly damage thyroid tissue through high-energy radiation, affecting not only tumor cells but also surrounding normal tissue, leading to thyroid cell damage or apoptosis and reduced hormone secretion. The immune system’s synergistic destructive effect occurs when immunotherapy activates the immune system, potentially triggering immune-mediated thyroiditis, while radiation-induced tissue damage increases thyroid susceptibility to immune attacks, resulting in more severe dysfunction. This is a primary reason for the objective results seen in phase 3 clinical trials of combined radiotherapy and immunotherapy. Radiation-induced fibrosis can replace normal thyroid tissue with scar tissue, further compromising thyroid function and hormone secretion. Chronic inflammation is enhanced by radiotherapy, particularly in the context of immunotherapy, affecting both thyroid function and recovery capacity, leading to more pronounced and persistent hypothyroid symptoms. The complexity of combination therapy increases the difficulty of individualizing treatment plans, and in some cases in this study, larger radiation doses and fields increased the risk of unnecessary thyroid damage, exacerbating hypothyroidism severity. Thus, the synergistic effect of radiotherapy’s direct physical damage, fibrosis, and immune-mediated injury from immunotherapy results in more severe hypothyroidism with higher incidence and severity rates.

In radiochemotherapy with immunotherapy, four patients progressed from subclinical hyperthyroidism to clinical hypothyroidism. This progression could result from either anti-PD-1 immunotherapy alone or combined effects with radiation therapy. “Normal” thyroid hormone levels during treatment may not represent true normal function, as clinical symptoms can still occur. Preventive levothyroxine is typically avoided to prevent potential hyperthyroidism. Management focuses on precise thyroid protection during radiation, optimized treatment timing, antioxidant supplementation, appropriate nutrition, and collaborative care between specialties for continuous monitoring.

Current limitations in this study of thyroid dysfunction following immunotherapy combined with radiochemotherapy for nasopharyngeal cancer include:1. Small sample size and short follow-up period, potentially underestimating late-onset thyroid dysfunction. 2. Irregular thyroid function monitoring in some patients. 3. Some patients received brief ICI treatment without subsequent thyroid monitoring, leading to inaccurate onset timing and missed thyroid irAE diagnoses. 4. Lack of thyroid ultrasound and antibody results, preventing comparison of thyroid volume and echo changes across different functional states. 5. Future studies could benefit from including FDG-PET/CT examination, which provides valuable systemic metabolic information for monitoring immune therapy response, distinguishing active tumors from pseudoprogression, and identifying checkpoint inhibitor-related adverse effects (28).

This report finds that nasopharyngeal cancer patients receiving immunotherapy combined with radiochemotherapy showed higher incidence and severity of thyroid dysfunction compared to those receiving immunotherapy with chemotherapy alone. The study’s limitations include its single-center nature with small sample size and potential selection bias. The lack of information about other subgroups, particularly those with different demographic or clinical characteristics, may affect the strength of associations. Additionally, the short follow-up period and center-specific patient population and institutional practices may impact results, reducing external validity. Future research should employ multi-center approaches with longer study periods to ensure more representative sampling and improve reliability of conclusions.

## Conclusions

NPC patients receiving anti-PD-1 immunotherapy combined with radiochemotherapy show increased incidence and severity of thyroid dysfunction. The risk of thyroid dysfunction is significantly higher in nasopharyngeal cancer patients who smoke, have thyroid nodules, and cervical lymph node metastases when receiving anti-PD-1 immunotherapy combined with IMRT.

## Data Availability

The raw data supporting the conclusions of this article will be made available by the authors, without undue reservation.
